# Hsa_circ_0005623 is an indicator for pulmonary artery hypertension associated with congenital heart disease

**DOI:** 10.3389/fcvm.2025.1561069

**Published:** 2026-01-12

**Authors:** Yuanhao Zhang, Yan Han, Zirui Sun, Weizhen Xing, Hao Tang, Chuanyu Gao, Yu Han

**Affiliations:** 1 Department of Structural Heart Disease, Central China Fuwai Hospital of Zhengzhou University, Zhengzhou, Henan, China; 2 National Health Commission Key Laboratory of Cardiovascular Regenerative Medicine, Central China Branch of National Center for Cardiovascular Diseases, Zhengzhou, Henan, China

**Keywords:** bioinformatic analyses, biomarker, circRNAs, congenital heart disease, pulmonary hypertension

## Abstract

There is a strong correlation between delayed diagnosis and high mortality rate in pulmonary arterial hypertension (PAH). Recent research indicates that circular RNAs (circRNAs) may serve as potential diagnostic biomarkers for PAH. This study aimed to identify important circRNAs associated with PAH to support early diagnosis and explore possible key disease mechanisms. GSE171827 and GSE113439 were obtained from the Gene Expression Omnibus (GEO) database to evaluate differentially expressed circular RNAs (DECs) and genes (DEGs). MicroRNAs (miRNAs) related to PAH were obtained from the Human microRNA Disease Database (HMDD). We validated changes in DEC expression levels using RT-qPCR in hypoxia- and normoxic-induced human pulmonary artery endothelial cells. Then, the potential relationship between DEC expression levels and mean pulmonary artery pressure (mPAP) in PAH patients was investigated. Finally, bioinformatics analyses were performed to construct a competing endogenous RNA (ceRNA) network and excavate the potential functions of DECs. Only hsa_circ_0005623 expression was significantly downregulated in PAH. Low hsa_circ_0005623 expression levels in the plasma of PAH patients were significantly associated with mPAP (*p* < 0.001). A ceRNA network comprising 1 circRNA (hsa_circ_0005623), 4 miRNAs (has-miR-424-5p, has-miR-503-5p, has-miR-331-3p, and has-miR-17-3p), and 10 mRNAs (CDH5, ANGPT2, DLL4, CLDN5, ANGPTL4, EDN1, HEY1, GATA2, CLEC14A, and ADM) was identified. Functional enrichment analysis of these 10 hub genes showed enrichment in endothelium development and blood vessel endothelial cell migration. These results suggest that hsa_circ_005623 in plasma is a potential biomarker for early PAH and may play an important role in the development of PAH.

## Introduction

Pulmonary arterial hypertension (PAH) is a chronic disease characterized by endothelial dysfunction and the proliferation of smooth muscle cells and fibroblasts in the pulmonary arteries. These changes lead to progressive stenosis of small pulmonary arteries, eventually resulting in right heart failure and death (1). Recognizing undiagnosed PAH is challenging in the primary care setting, with a mean time to diagnosis of 2 years (2). The predominant cause of delayed diagnosis is the non-specific clinical symptoms and insidious progression of the disease. Common symptoms like dyspnea and fatigue require differential diagnosis from conditions including heart failure, coronary atherosclerotic heart disease (CAHD), chronic obstructive pulmonary disease (COPD), and pulmonary fibrosis.

In clinical practice, the diagnosis of PAH necessitates combining patient history, echocardiography, chest radiography, and electrocardiography. Basic laboratory testing is also needed. Currently, there is no validated PAH-specific biomarker for diagnosis; however, brain natriuretic peptide (BNP) is routinely measured for risk stratification in patients with PH (3). Right heart catheterization (RHC) remains the gold standard for diagnosing and classifying PAH; however, its invasive nature and relatively high cost significantly limit its use for repeated assessments in clinical practice.

A common cause of PAH is a left-to-right shunt in congenital heart disease (CHD). Studies have shown that approximately 67% of pediatric PAH cases are associated with CHD (PAH-CHD) (4). Even in adult CHD patients, the prevalence of PAH is as high as 5%–10% (5). Surgical repair or percutaneous transcatheter defect occlusion are common treatments for CHD. However, a significant proportion of patients miss the intervention treatment opportunities because the delayed diagnosis allows PAH to progress to an advanced stage. Current drugs for PAH, such as endothelin receptor antagonist (ERA) and soluble guanylate cyclase (sGC), provide symptomatic relief but do not cure the disease. Therefore, identifying new targets for early diagnosis is meaningful.

The competitive endogenous RNA (ceRNA) hypothesis proposes that ceRNAs (mostly lncRNA and circRNA) participate in transcriptional and posttranscriptional regulation through mechanisms such as microRNA (miRNA) response element-mediated regulation of mRNA stability (6). CircRNAs are generated by back-splicing of exons from precursor mRNA in eukaryotes (7). As natural miRNA sponges, circRNAs have been found to play an important role in lung cancer and asthma (8, 9) and may serve as potential diagnostic biomarkers and therapeutic targets. However, the role of circRNAs in PAH-CHD remains unclear.

**Figure 1:**
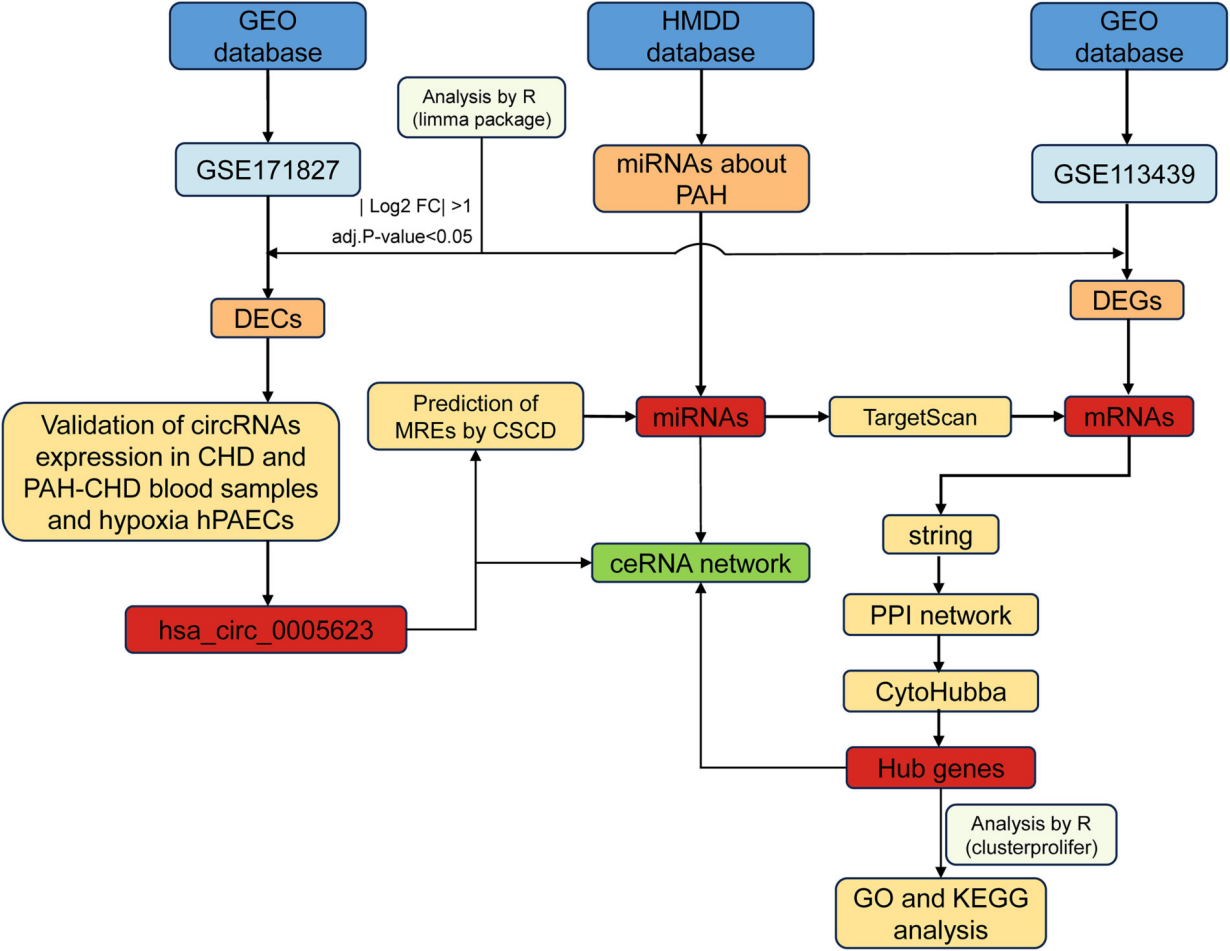
Flowchart of this study.

In this study, we screened and identified differentially expressed circular RNAs (DECs) associated with PAH and constructed a circRNA–miRNA–mRNA network. We also validated changes in DEC expression levels in hypoxia-induced human pulmonary artery endothelial cells (hPAECs), suggesting that they may play an important role in the development of PAH. The methodology underlying the bioinformatics analysis and validation is shown in Figure 1.

## Materials and methods

### Data collection and preprocessing

We used “pulmonary hypertension” as a keyword to retrieve two datasets—GSE171827 (circRNA expression dataset) and GSE113439 (mRNA expression dataset)—from the NCBI GEO database [http://www.ncbi.nlm.nih.gov/geo/ (accessed on December 20, 2024)] (10). Among the various subtypes of PH, PAH-CHD was selected for further analysis. These datasets were subjected to background correction, normalization, and calculation of expression values using the robust multiarray average (RMA) normalization method implemented in the “Affy” library (version 1.84.0) (11). Probes with missing expression values and genes of unknown function were removed. Probe names were converted to gene symbols using the platform annotation information, and the average expression value of multiple probes corresponding to one gene was considered as the expression value of this gene. Principal component analysis (PCA) was used to find similarities between samples. All preprocessing steps were performed using R software (R version 4.4.1).

### Differential expression analysis

The “Limma” package (version 3.52.4) (12) was utilized to screen out DECs and differentially expressed genes (DEGs), and a |log2 fold change| > 1 and an adj. *p*-val < 0.05 were considered statistically significant. Volcano plots were mapped using the “ggplot2” package (13). Information and loop structure data about DECs were retrieved from circBase (14) (http://www.circbase.org/) and CSCD (15) databases (http://gb.whu.edu.cn/CSCD/).

### Formation of the circRNA/miRNA/mRNA regulatory network

The CSCD database was also used to predict miRNA response element (MRE) sites for each DEC. PAH-related miRNAs were obtained from the Human microRNA Disease Database (HMDD) (16). To acquire potential target miRNAs of the DECs, an intersection was performed between the predicted miRNAs and PAH-related miRNAs. In addition, TargetScan (http://www.targetscan.org/vert_72) (17) was used to predict mRNAs targeted by the miRNAs obtained from the previous step. These predicted mRNAs were then compared with the DEGs to identify candidate target mRNAs through intersection analysis. Finally, a ceRNA network comprising DECs, target miRNAs, and mRNAs was constructed and visualized using Cytoscape software (18).

### Functional enrichment analysis of target genes

Gene Ontology (GO) analysis is commonly used to investigate the biological attributes of genes and gene products, providing insights into the biological processes (BPs), cellular components (CCs), and molecular functions (MFs). We performed GO analysis using the “org.Hs.eg.db” (V 3.15.0) (19) package, with an adj. *p*-val < 0.05 considered statistically significant. We utilized the “ggplot2” package in R to visualize the results of the enrichment analysis.

### PPI network construction

To investigate potential protein–protein interactions (PPIs) among the identified target genes, we utilized the STRING (20) database (https://string-db.org/). PPI pairs with a combined score of at least 0.4 were considered to be biologically relevant. Then, we used Cytoscape software to visualize the network. The top 10 hub genes were found using CytoHubba (21), a Cytoscape plugin that applies 11 scoring systems to evaluate the significance of nodes in a biological network.

### Participants and plasma samples

In this study, all medical record data were fully anonymized prior to access. As a retrospective study, it was approved by the Ethics Committee of Central China Fuwai Hospital of Zhengzhou University (approval number: FZX-LUNL1-20240013), and the requirement for informed consent was waived. The study adhered to the ethical standards of the Declaration of Helsinki and its subsequent amendments. Between September 2022 and May 2024, 50 patients with CHD were recruited. Patients were classified into either the PAH-CHD group (mPAP > 20 mmHg) or the CHD group (mPAP ≤ 20 mmHg) based on their mPAP. The inclusion criteria were as follows: (1) echocardiography-confirmed left-to-right shunt CHD and (2) age between 18 and 80 years. Patients with other types of PH, poorly controlled hypertension or arrhythmia, severe liver/kidney dysfunction, critical infection, or malignancy were excluded. Patient blood samples were collected within 24–48 h of hospital admission. Venous blood (3–5 mL) was drawn from each participant and centrifuged (3,000 rpm, 4°C, 10 min), and the supernatant plasma was stored at −80 °C.

### hPAEC culture

HPAECs were obtained from ScienCell (Shanghai, China) and cultured in endothelial cell medium (ECM) at 37 °C with 5% CO_2_ and 95% relative humidity. The only difference between the control group and the hypoxia group was oxygen concentration: the control group was cultured at 21% O_2_, while the hypoxia group was cultured at 4% O_2_. All experiments were repeated at least three times.

### Quantitative reverse transcription and real-time PCR

Total RNA was isolated from plasma and hPAECs using TRIzol Reagent (Vazyme, Jiangsu, USA) and then reverse-transcribed into cDNA using a reverse transcription (RT) Premix for qPCR (Accurate, Hunan, China). Genomic DNA (gDNA) was extracted using the TIANamp Genomic DNA Kit (Tiangen Biotech). Real-time quantitative reverse transcription-polymerase chain reaction (RT-qPCR) was performed using GoTaq qPCR Master Mix (Promega). GAPDH was used to normalize the circRNA level. Relative expression of genes was measured using the 2^−△△Ct^ method. Each experiment was repeated at least three times independently. The primer sequences used for RT-qPCR are as follows (F, forward; R, reverse): GAPDH: F: 5′-CAGGAGGCATTGCTGATGAT-3′ and R: 5′-GAAGGCTGGGGCTCATTT-3′; hsa_circ_0005623: F: 5′-CCTTTGCAGAAGTCACCGGG-3′ and R: 5′-CCCAGGAGACCACAAAGCTAC-3′. For RT-qPCR analysis, a single-blind approach was adopted to minimize subjective bias.

### Statistical analysis

Data were statistically processed using R (4.4.1) and GraphPad Prism 9. Differential analysis of public datasets was performed using the R package “Limma” (3.52.4), and “ggplot2” was used for visualizing the results. Receiver operating characteristic (ROC) curves were generated using GraphPad Prism 9 to assess the diagnostic values. Correlation was calculated using Spearman correlation analysis. *P* < 0.05 was deemed statistically significant.

## Results

### Hsa_circ_0005623 expression was downregulated in PAH-CHD

**Figure 2:**
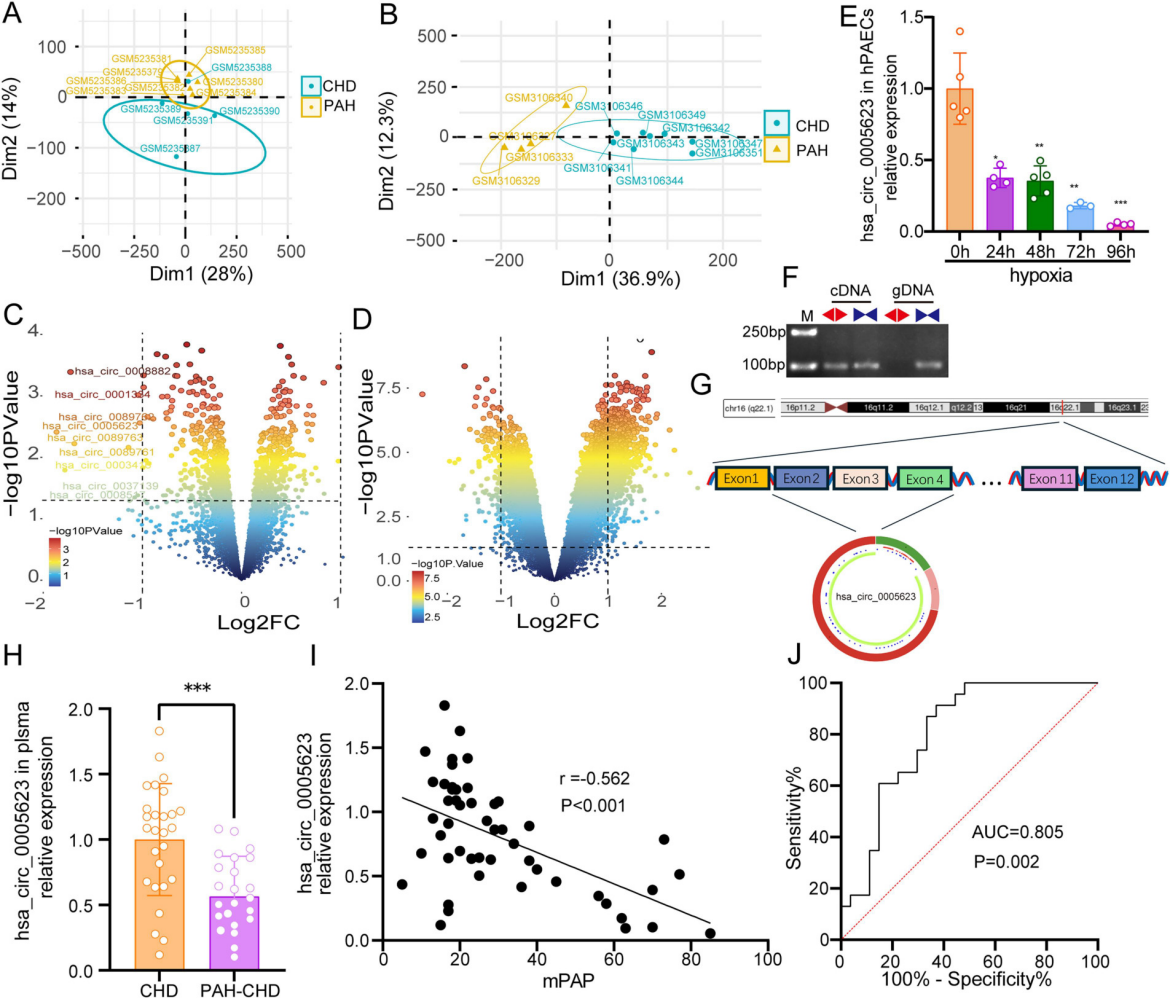
Validation of hsa_circ_0005623. **(A)** PCA clustering results of GSE171827: one of the samples was removed from the CHD group due to being spatially far from other CHD samples. **(B)** PCA clustering results of GSE113439. **(C,D)** Volcano plots of DECs and DEGs, respectively. The right and left dots represent upregulated and downregulated RNAs, respectively, with significance, which are distributed above the line representing *p* < 0.05 (adj. *p*-val < 0.05 and |log_2_FC| > 1). **(E)** Relative hsa_circ_0005623 expression levels in the hypoxia-induced hPAECs. **(F)** Divergent primers (◄►) for detecting hsa_circ_0005623 in complementary DNA (cDNA) but not in genomic DNA (gDNA). **(G)** Structural pattern of hsa_circ_0005623. **(H)** Relative hsa_circ_0005623 expression levels, which are lower in the plasma of patients with PAH-CHD than in those with CHD. **(I)** Expression level of hsa_circ_0005623, which is significantly negatively correlated with mPAP. **(J)** Receiver operating characteristic (ROC) curve of plasma hsa_circ_0005623.

After dimensionality reduction by PCA of the data and assessment of similarities between sample groups, GSE171827 included six PAH-CHD and four CHD cases, while GSE113439 contained eight PAH-CHD and four CHD cases (Figures 2A,B). As shown in Figure 2C, nine circRNAs were significantly downregulated in PAH-CHD compared with CHD. In addition, 810 and 138 mRNAs were significantly upregulated and downregulated in PAH-CHD, respectively (Figure 2D). Then, we found that hsa_circ_0005623 expression was significantly downregulated in hypoxia-induced hPAECs, and the downregulation became more pronounced with increasing hypoxia duration (Figure 2E). Divergent primers only amplified hsa_circ_0005623 in cDNA samples but not in gDNA, indicating the presence of the back-spliced junction of hsa_circ_0005623 (Figure 2F). To gain further insights into hsa_circ_0005623, we used the CSCD database to describe its structural loop graphs. According to the circBase database, hsa_circ_0005623 (chr16:68300495–68309152) is derived from exons 2, 3, and 4 of SLC7A6 gene and is 774 base pairs in length (Figure 2G). The expression levels of hsa_circ_0005623 in plasma were measured in 27 CHD and 23 PAH-CHD participants by qRT-PCR and were significantly lower in the PAH-CHD group than in the CHD group (*p* < 0.001) (Figure 2H). We also found that the expression levels of hsa_circ_0005623 were significantly associated with mPAP, with expression decreasing as mPAP increased (*r* = −0.562, *p* < 0.001) (Figure 2I). ROC analysis showed the potential diagnostic capability of plasma hsa_circ_0005623; the area under the curve (AUC) of plasma hsa_circ_0005623 was 0.805 (95% confidence interval = 0.683–0.927, *p* = 0.002) (Figure 2J).

### Hub gene identification and enrichment analyses

**Figure 3:**
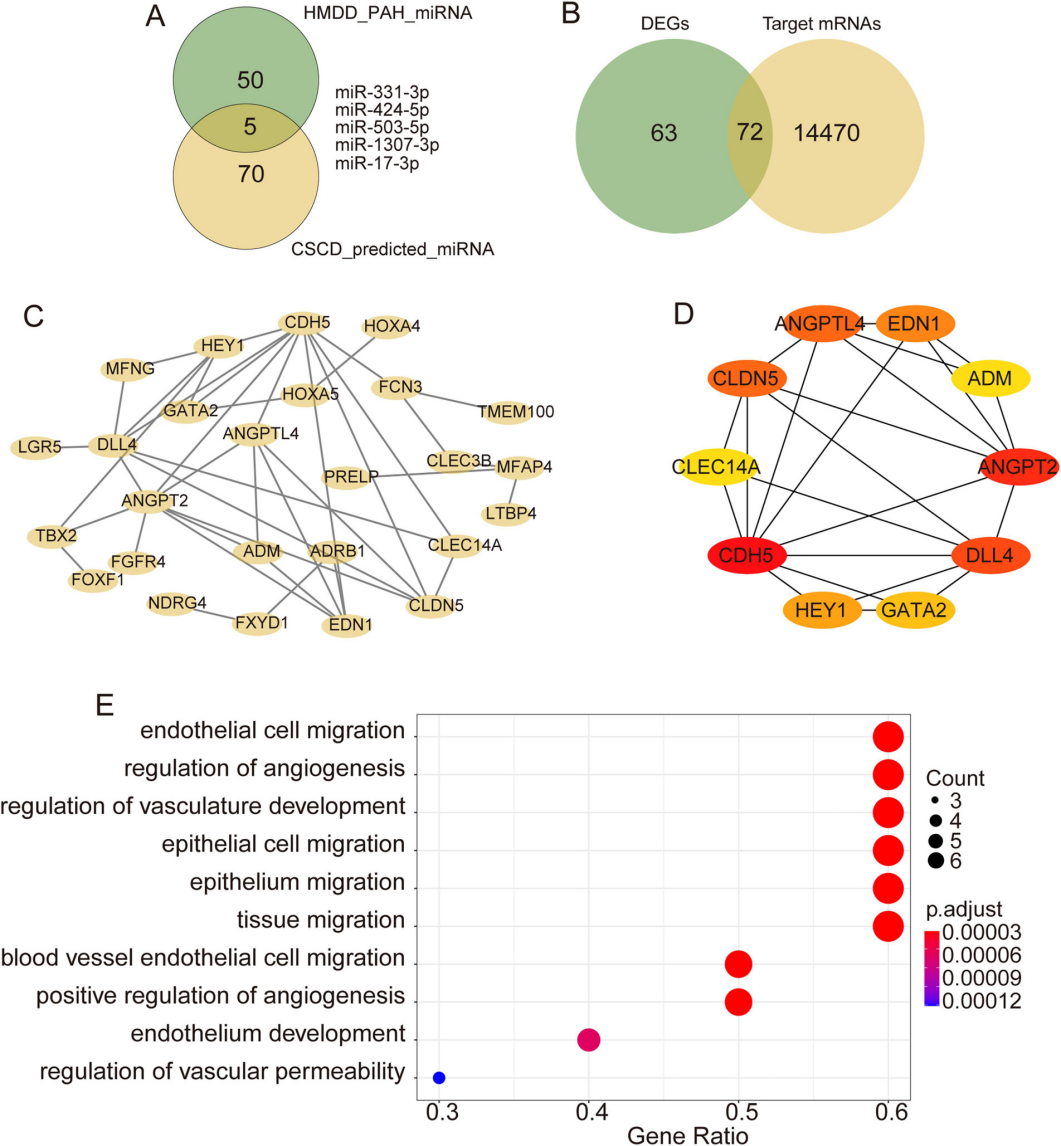
Bioinformatics analysis of hsa_circ_0005623. **(A)** Intersection analysis of target miRNAs of hsa_circ_0005623 and miRNAs related to PH from HMDD. **(B)** Intersection analysis of target mRNAs of candidate miRNAs and DEGs. **(C)** PPI networks of target genes constructed based on the STRING database. **(D)** Top 10 hub genes identified using CytoHubba. **(E)** GO enrichment analyses of the hub genes.

After intersecting the predicted miRNAs from CSCD with PAH-related miRNAs, five miRNAs targeting hsa_circ_0005623 were identified (Figure 3A). TargetScan was then used to predict mRNAs involved in the miRNA–mRNA interaction pair, uncovering 14,542 mRNAs. Intersection of these prediction results with the DEGs resulted in 72 candidate mRNAs (Figure 3B). A PPI network was constructed using the STRING database and visualized using Cytoscape software (Figure 3C). We identified the top 10 hub genes (CDH5, ANGPT2, DLL4, CLDN5, ANGPTL4, EDN1, HEY1, GATA2, CLEC14A, and ADM) in the network using the CytoHubba plugin (Figure 3D). GO analysis was performed to predict the potential functions of these 10 hub genes. Functions in biological process (BPs) were mainly enriched in endothelial cell migration, regulation of angiogenesis, and regulation of vasculature development (Figure 3E).

### Construction of a ceRNA network involving 1 circRNAs, 4 miRNAs, and 10 mRNAs

**Figure 4:**
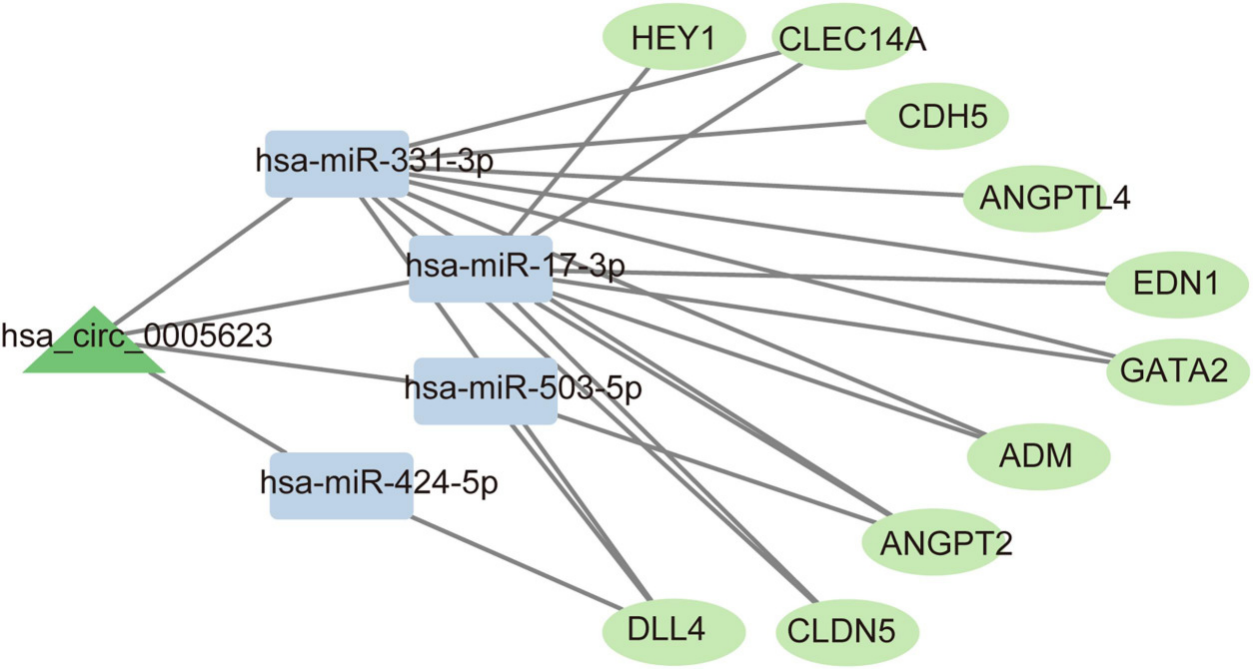
Interaction network map of the circRNA–miRNA–target gene axis.

Based on the hub genes, the circRNA–miRNA–mRNA network comprising 1 circRNAs (hsa_circ_0005623), 4 miRNAs (has-miR-424-5p, has-miR-503-5p, has-miR-331-3p, and has-miR-17-3p), and 10 mRNAs (CDH5, ANGPT2, DLL4, CLDN5, ANGPTL4, EDN1, HEY1, GATA2, CLEC14A, ADM) was constructed (Figure 4).

## Discussion

In this study, differential expression analysis and RT-qPCR validation revealed that hsa_circ_0005623 is significantly downregulated in the plasma of patients with PAH-CHD. Its expression level was significantly negatively correlated with mPAP and can be a specific indicator for PAH. By predicting targets, constructing a PPI network, and screening hub genes, we constructed a ceRNA network. Functional enrichment analysis of the network mRNAs highlighted pathways related to endothelial cell proliferation and migration, which is consistent with the downregulation of hsa_circ_0005623 in hypoxia-induced hPAECs. This suggests that hsa_circ_0005623 may not only serve as a diagnostic marker but also play a regulatory role in PAH development.

PAH is often termed “the cancer” of cardiovascular diseases, with an estimated global prevalence of 1% (22). Its pathophysiology involves multifactorial mechanisms, and clinical symptoms progress insidiously and lack specificity, often leading to delayed diagnosis and poor prognosis (23). With advances in targeted therapies, including those targeting the endothelin, prostacyclin, and nitric oxide (NO) pathways, the prognosis of PAH has significantly improved. A Chinese multicenter cohort study demonstrated significant survival improvement in idiopathic PAH (IPAH), with 3-year survival rates increasing from 48% to 75.1% (24). Nevertheless, patients continue to face high treatment costs and frequent hospitalizations. Current pharmacological therapies relieve symptoms but lack proven disease-modifying effects, failing to halt progression or improve long-term survival.

PAH-CHD, a subgroup of pulmonary hypertension (PH), arises from congenital systemic-to-pulmonary shunts. This condition predominantly affects small pulmonary arteries distal, characterized by dysfunctional proliferation of pulmonary arterial endothelial and smooth muscle cells. In developing nations such as China, the emergence and progression of PAH render surgical correction contraindicated in a substantial proportion of CHD patients (25). RHC remains the gold standard for diagnosing and classifying PAH. However, its invasive nature and substantial costs restrict clinical application, particularly for early detection and longitudinal monitoring. Given the diagnostic challenges, therapeutic limitations, and adverse prognosis associated with PH, developing accessible screening methods for early detection remains crucial. Particularly in PAH-CHD, disease progression is time-dependent, and timely intervention targeting the defect may prevent irreversible disease advancement.

circRNAs are non-coding RNAs. Reverse splicing forms a closed-loop structure that makes circRNAs highly stable. Most circRNAs are derived from exon sequences of precursor mRNAs (26). Current research indicates that circRNAs can regulate the transcription and splicing of host or non-host genes, modulate the stability and translation of cytoplasmic mRNAs, and interfere with signaling pathways (27). Their biological roles include binding to RNA polymerase II (Pol II) (28), recruiting proteins to specific regions of target promoters (29), binding to miRNAs to regulate mRNA expression or stability (6, 30), and interacting with RNA-binding proteins (31). Increasing evidence suggests that circRNAs play a crucial role in pulmonary vascular remodeling in PAH. For example, circ-calm4 has been found to regulate PASMC proliferation by modulating cell cycle progression, and its knockdown inhibited hypoxia-induced increase in PASMC proliferation and improved hypoxia-induced PH characteristics in mice (32). Other circRNAs, including hsa_circ_0002062 (33), CircATP2B4 (34), and circ-Grm1 (35), have also been reported to play important roles in the proliferation and migration of PASMCs and PAECs. Current research evidence suggests that circRNAs may serve as diagnostic biomarkers and therapeutic targets for pulmonary vascular remodeling by regulating the function of PASMCs and PAECs in PAH-CHD.

Bioinformatic analysis identified nine downregulated circRNAs in PAH-CHD. Through a review of the literature, we found that hsa_circ_0003416 exhibited significant downregulation, demonstrating its potential as a diagnostic biomarker for PAH-CHD (36, 37).

Given the scarcity of clinical specimens, we first conducted *in vitro* experiments for initial validation. Hypoxia-exposed hPAECs and hPASMCs are widely employed experimental models for studying PH mechanisms (38). Considering the influence of hemodynamics on endothelial RNA profiles (39), we first verified the changes in DEC expression in hPAECs under hypoxic conditions. Hsa_circ_0005623 showed progressive downregulation with increasing hypoxia duration, consistent with the bioinformatic findings. Further literature review revealed that hsa_circ_0005623 originates from the SLC7A6 locus, which encodes an L-arginine transporter essential for nitric oxide synthase activity (40, 41). This suggests a potential role for hsa_circ_0005623 in regulating endothelial function through modulation of the arginine–NO pathway during PAH progression, although further functional validation is necessary.

RNA extracted from patient blood samples confirmed significant downregulation of hsa_circ_0005623 in PAH-CHD. Correlation analysis demonstrated a negative correlation between its plasma expression levels and mPAP. ROC curve analysis further supported its potential as a diagnostic biomarker for PAH-CHD. The construction of a ceRNA network based on the prediction of circRNA–miRNA interactions represents a well-established bioinformatics approach (42). In this investigation, we applied a novel screening strategy to identify PH-associated miRNAs via database integration, followed by the prediction of their target genes and intersection with DEGs, resulting in the identification of 10 hub genes. This systematic methodology strengthens the pathophysiological relevance of our ceRNA network to PAH. Functional enrichment analysis of the hub genes revealed significant associations with BPs critical to vascular homeostasis, including endothelial cell migration, angiogenesis regulation, and vascular development. These findings align with the established pathophysiological features of PAH, which is characterized by aberrant endothelial proliferation, migratory dysregulation, and phenotypic switching. The observed downregulation of hsa_circ_0005623 in endothelial cells further supports its potential involvement in PAH pathogenesis through modulation of vascular remodeling.

Ten key genes (CDH5, ANGPT2, DLL4, CLDN5, ANGPTL4, EDN1, HEY1, GATA2, CLEC14A, and ADM) were reported in this study. Most of them have been associated with angiogenesis or vascular endothelial function, as indicated by the results of current studies. For example, CDH5 is a transmembrane glycoprotein that plays a key role in endothelial cell migration, apoptosis, intercellular adhesion, and contact inhibition of growth. The current study suggests that upregulation of CDH5 expression is associated with neovascularization (43), whereas downregulation of CDH5 is closely linked to endothelial dysfunction (44). Due to the specific expression of CDH5 in endothelial cells, CDH5-Cre mice are currently used to specifically knock down target genes in vascular endothelial cells. As another example, ANGPTL4 belongs to the angiopoietin-like protein family, whereas ANGPT2 is a member of the angiopoietin family. Although they share similar structural domains, these two proteins exhibit different characteristics in terms of gene expression and function. ANGPT2 is mainly produced by endothelial cells and is expressed at low levels in normal tissues but is usually highly upregulated in the tumor-associated vasculature (45). Numerous studies have shown that ANGPT2 increases vascular instability, and higher ANGPT2 levels are associated with poorer survival in PAH patients (46, 47). However, it has also been reported that downregulation of ANGPT2 can affect endothelial cell function to some extent (48). In contrast, ANGPTL4 is highly expressed in human adipose tissue and liver and is involved in various physiological processes, such as angiogenesis, glycolipid metabolism, and inflammation (49). Elevated ANGPTL4 expression has been observed in the serum of patients with heart failure with reduced ejection fraction (HFrEF) and COPD and has been shown to be independently correlated with NT-proBNP (50, 51). Thus, serum ANGPTL4 may, to some extent, reflect cardiovascular function. Another study found that cardiomyocyte hypertrophy and endothelial injury were exacerbated after downregulation of ANGPTL4 (52). Further research may be needed to understand these conflicting findings. The other genes have also been reported to be associated with PAH or endothelial cells. These findings indicate that the ceRNA network we constructed is closely related to PAH, and validating its underlying pathways will be the next focus of our research group.

However, we have to acknowledge the limitations of this study. For example, the development of PAH-CHD is influenced by both environmental and genetic factors, and future research should consider evaluating these and other potential contributing factors, such as environmental risk factors and family history of PAH-CHD. Further evidence is needed to fully understand the biological basis of these genes in the development of PAH-CHD. In subsequent investigations, we plan to perform *in vitro* validation of pathway-associated RNA interactions and elucidate their mechanistic contributions to PAH pathogenesis.

## Conclusion

We found that hsa_circ_0005623 may serve as a potential diagnostic biomarker for PAH-CHD. We also constructed a ceRNA network of PAH-CHD comprising one circRNA, five miRNAs, and 10 mRNAs. These genes may provide new points for further research on PAH-CHD.

## Data Availability

The original contributions presented in the study are included in the article/Supplementary Material, further inquiries can be directed to the corresponding authors.
